# Impaired Innate COPD Alveolar Macrophage Responses and Toll-Like Receptor-9 Polymorphisms

**DOI:** 10.1371/journal.pone.0134209

**Published:** 2015-09-11

**Authors:** Charles S. Berenson, Ragina L. Kruzel, Catherine T. Wrona, Manoj J. Mammen, Sanjay Sethi

**Affiliations:** 1 Division of Infectious Diseases, Department of Veterans Affairs Western New York Healthcare System, State University of New York at Buffalo School of Medicine, Buffalo, New York, 14215, United States of America; 2 Division of Pulmonary, Critical Care, and Sleep Medicine, Department of Veterans Affairs Western New York Healthcare System, State University of New York at Buffalo School of Medicine, Buffalo, New York, 14215, United States of America; 3 Department of Biomedical Informatics, State University of New York at Buffalo School of Medicine, Buffalo, New York, 14215, United States of America; Louisiana State University, UNITED STATES

## Abstract

**Background:**

Dysfunctional innate responses of alveolar macrophages to nontypeable *Haemophilus influenzae*, *Moraxella catarrhalis* and *Streptococcus pneumoniae* contribute to morbidity in chronic obstructive pulmonary disease (COPD). Our earlier studies discovered impaired COPD alveolar macrophage responses to Toll-like receptor (TLR) ligands of nontypeable *H*. *influenzae* and provide rationale for further evaluation of TLR signaling. While the role of TLR single nucleotide polymorphisms is increasingly recognized in inflammatory diseases, TLR single nucleotide polymorphisms in COPD have only recently been explored. We hypothesized that specific TLR polymorphisms are associated with dysfunctional innate immune COPD alveolar macrophage responses and investigated polymorphisms of TLR2(Arg753Gln), TLR4(Thr399Ile; Asp299Gly), and TLR9(T1486C; T1237C).

**Methods:**

DNA was purified from cells of 1) healthy nonsmokers (n = 20); 2) COPD ex-smokers (n = 83); 3) COPD active smokers (n = 93). DNA amplifications (polymerase chain reaction) were performed for each SNP. Alveolar macrophages from each group were incubated with nontypeable *H*. *influenzae*, *M*. *catarrhalis* and *S*. *pneumoniae*. Cytokine induction of macrophage supernatants was measured and the association with TLR single nucleotide polymorphism expression was determined.

**Results:**

No significant inter-group differences in frequency of any TLR SNP existed. However both TLR9 single nucleotide polymorphisms were expressed in high frequency. Among COPD ex-smokers, diminished IL-8 responsiveness to nontypeable *H*. *influenzae*, *M*. *catarrhalis* and *S*. *pneumoniae* was strongly associated with carriage of TLR9(T1237C) (p = 0.02; p = 0.008; p = 0.02), but not TLR9(T1486C). Carriage of TLR9(T1237C), but not TLR9(T1486C), correlated with diminished FEV_1_%predicted (p = 0.037).

**Conclusion:**

Our results demonstrate a notable association of TLR9(T1237C) expression with dysfunctional innate alveolar macrophage responses to respiratory pathogens and with severity of COPD.

## Introduction

Bacterial colonization of the airways in COPD has a dynamic interaction with host immune factors that is tied to advancement of disease [1,2]. Once initiated, chronic inflammation and impaired immunologic responses may fail to clear bacteria of lower airways and promote progression of COPD [3,4]. Alveolar macrophages in COPD have strikingly dysfunctional responses to TLR2 and TLR4 ligands of nontypeable *H*. *influenzae* (NTHI), as well as impaired phagocytosis of NTHI [5,6].

Recent studies indicate that phagocytic impairment of COPD alveolar macrophages is not limited to NTHI, but extends to *M*. *catarrhalis* (MC), yet not to inert particles, demonstrating a pathogen-associated molecular pattern (PAMP)-mediated, complement-independent defect [7]. Alveolar macrophages of COPD active smokers also had diminished phagocytosis. However, they had less impairment of pathogen-mediated IL-8 induction than alveolar macrophages of COPD ex-smokers. There was a direct correlation between the severity of COPD and the degree of impaired alveolar macrophage phagocytosis for both NTHI and MC. Yet, impaired pathogen-mediated IL-8 induction correlated with a propensity toward COPD exacerbations [8]. Collectively, these studies support a hypothesis that dysfunctional alveolar macrophage responses toward respiratory pathogens contribute to chronic airway persistence of bacteria and to decline of function in COPD. Although our investigations have revealed the importance of PAMP recognition and complement-independence, underlying host factors have only been partly explored.

Impaired COPD alveolar macrophage responses to TLR2 and TLR4 ligands [9,10] provide rationale for further evaluation of TLR signaling. Polymorphisms of TLRs in humans [9,11] may result in diminished inflammation and increased susceptibility to Gram-negative infections [9,11]. For example, healthy volunteers, heterozygous for TLR4 SNPs, had lower systemic inflammatory responses to inhaled LPS than did wildtype counterparts [12,13]. Attenuated receptor signaling through TLR SNPs may affect a broad range of diverse biologic conditions [11,14].

Recent study has begun to focus on the potential contribution of TLR polymorphisms to pulmonary diseases. Associations of TLR SNPs have been sought, not only with COPD [15–17], but with a variety of respiratory conditions [18–20]. We hypothesized that select TLR polymorphisms are associated with dysfunctional innate alveolar macrophage responses in COPD and performed experiments to test this hypothesis.

## Materials and Methods

### Ethics statement

All procedures received approval from the Veterans Affairs Western New York Institutional Review Board. Written informed consent was obtained from all volunteers.

### Recruitment of subjects

Volunteers were screened for inclusion into: Group 1: nonCOPD nonsmokers; Group 2: ex-smokers with COPD; Group 3: active smokers with COPD. All participants were over 30 years of age and underwent clinical assessment, routine spirometry and chest x-rays. Ex-smokers and healthy nonsmokers had expired breath carbon monoxide of <0.02ppm (Vitalograph Breath CO Monitor, Lenexa, KS). No participants were taking systemic steroids or antibiotics.

For COPD participants, exclusion criteria included a forced expiratory volume at one second (FEV_1_) <35%, hypercapnia and co-morbid diseases that would render bronchoscopy unsafe. Inclusion criteria were: 1) chronic bronchitis by history and/or emphysema by chest x-ray or CT; 2) absence of other lung disease, including asthma and bronchiectasis, based on clinical evaluation; 3) chest x-ray findings that were normal or compatible with COPD, but detected no other disease; 4) FEV_1_/FVC ratio below the 95% lower confidence limit of normal on spirometry; 5) non-atopic by history; 6) no antibiotic or systemic steroid use for four weeks preceding enrollment. Healthy non-smokers had never smoked and met all inclusion criteria of the COPD group, except #1 and #4 (above) [5].

### Purification of human alveolar macrophages

Participants underwent bronchoalveolar lavage (BAL) to obtain alveolar macrophages, which were purified by density gradient centrifugation and plated, as previously detailed [5,6]. After incubation, non-adherent cells were removed. Remaining alveolar macrophages were 98–100% esterase-positive and 88–90% viable. Percent viability of alveolar macrophages (mean±SEM), by trypan blue exclusion, was 89.1±1.2, 88.7±0.9 and 89.6±.7 for Groups 1, 2 and 3 respectively (p>0.1).

### DNA isolation

Polymerase chain reaction (PCR) analysis of gene mutations was performed on sterile 10 ml. venous blood samples, from each participant. Genomic DNA was isolated using commercially available reagents (Wizard genomic DNA purification kit, Promega Corp., Madison WI).

#### Determination of TLR polymorphisms

**Fig 1:**
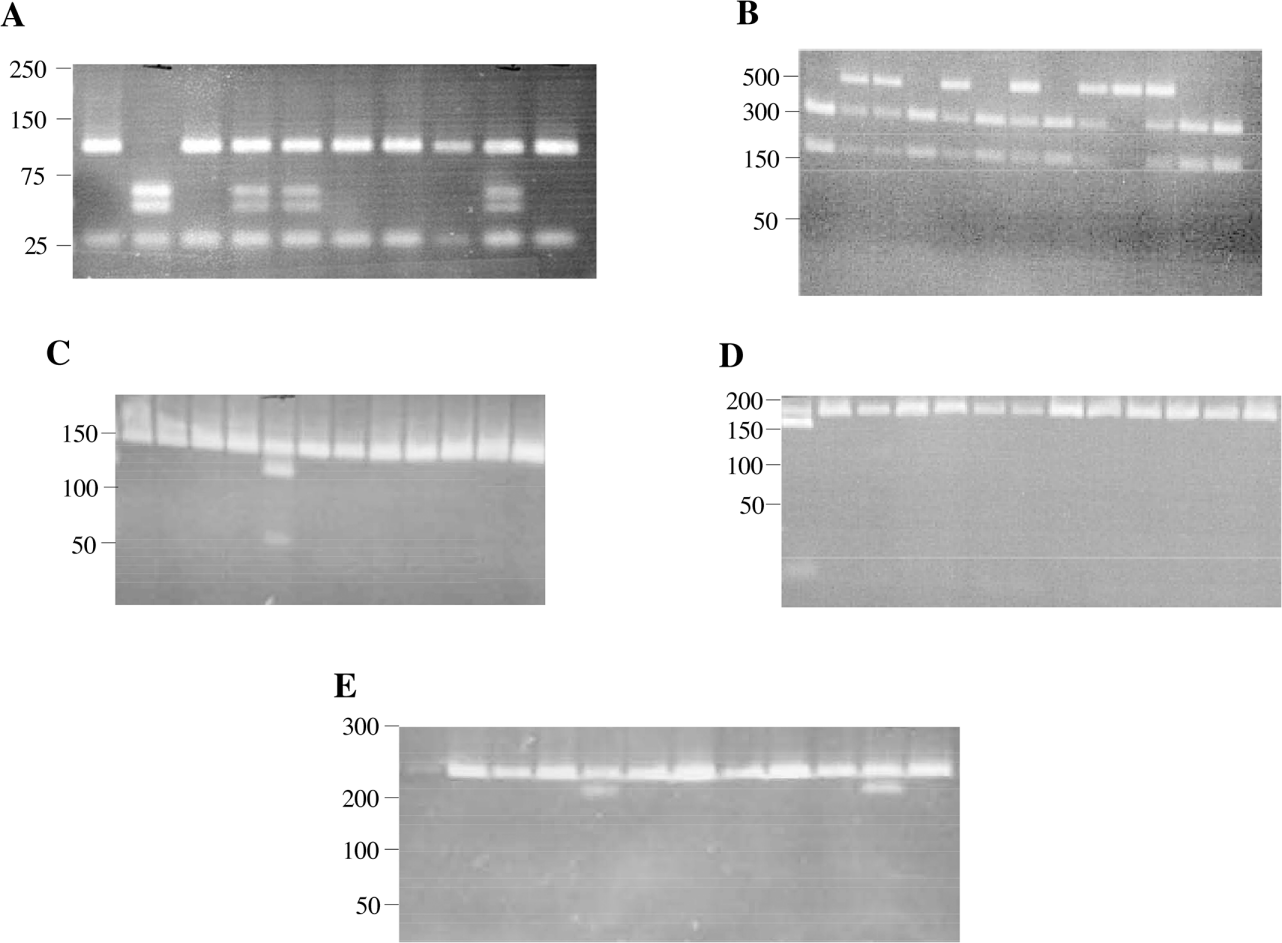
Single nucleotide polymorphisms of TLR’s. PCR-based RFLP’s were performed with genomic DNA from each participant. Representative agarose gels of shown for: TLR9 (T1237C) (Panel A), TLR9 (T1486C) (Panel B), TLR4 (Thr399Ile) (Panel C), TLR4 (Asp299Gly) (Panel D) and TLR2 (Arg753Gln) (Panel E). Numbers in left margin of each gel correspond to DNA ladder base pairs. Lanes with restriction fragments for heterozygotes and homozygotes of each SNP are distinguishable from restriction fragments for wildtypes. Representative patient samples are shown in each lane of each panel.

SNPs of each DNA sample were determined by PCR-based restriction fragment length polymorphism (RFLP). PCR was carried out in a 25–30 ul mixture containing 1 ug of genomic DNA, Go Taq Green Master Mix (400uM of dATP, dCTP, dTTP, dGTP, Taq polymerase, 3mM MgCl_2_; Promega Corp., Madison WI) and 1ul each of 0.1uM primer (Sigma Chemical Co., St. Louis MO), detailed on Table 1. PCR was performed in a thermocycler with denaturation and annealing steps, varied for each TLR polymorphism following published methods [21–23]. 3ul of PCR products were digested overnight and mixed with 7ul of restriction enzyme (New England Biolabs Inc., Ipswich, MA) for each (Table 1). 10 ul of PCR product was loaded on agarose gels, varied for each TLR polymorphism according to published methods [24–26] including 1ul loading buffer (EMD Chemicals, San Diego, CA) (Fig 1). Representative patient samples are shown in each lane of each panel of Fig 1. DNA quantity was insufficient for analysis for some samples of each group. For clarity, each SNP designation is also provided in Table 1 as given in the public single-nucleotide polymorphism database (dbSNP).

**Table 1:** Nucleotide sequences of synthetic oligonucleotides for PCR-based RFLP for single nucleotide polymorphisms.

TLR polymorphism		Primer sequence	PCR Product	Restriction Enzymes
TLR2 (Arg753Gln)	rs5743708*	F: 5’GGGACTTCATTCCTGCAAGT3’	264bp	SfcI
		R: 5’GGCCACTCCAGGTAGGTCTT3’		
TLR4 (Asp299Gly)	rs4986790*	F: 5’AGCATACTTAGACTACTAAATC3’	188bp	NcoI
		R: 5’GAGAGATTTGAGTTTCAATGTG3’		
TLR (Thr399Ile)	rs4986791*	F: 5’GGTTGCTGTTCTCAAAGTGATTTTGGGAGAA3’	124bp	HinfI
		R: 5’GGAAATCCAGATGTTCTAGTTGTTCTAAGCC3’		
TLR9 (Thr1486Cys)	rs5743836*	F: 5’TCCCAGCAGCAACAATTCATTA3’	499bp	Afl II
		R: 5’CTGCTTGCAGTTGACTGTGT3’		
TLR9 (Thr1237Cys)	rs187084*	F: 5’ATGGGAGCAGAGACATAATGG3’	135bp	BstN1
		R: 5’CTGCTTGCAGTTGACTGTGT3’		

* SNP designation in the Single Nucleotide Polymorphism Database (dbSNP)

### Macrophage-bacteria incubation

To establish the proinflammatory effect of individual respiratory pathogens, each donor’s alveolar macrophages were inoculated with: nontypeable *Haemophilus influenzae* 11P6H1; *Streptococcus pneumoniae* 25P55 and *Moraxella catarrhalis* 6P29B1. Details on characterization of bacterial strains are given in (S1 Text). Bacteria were incubated with adherent alveolar macrophages at a multiplicity of infection (macrophages:bacteria) of 1:200. Supernatants were harvested at 6 hours, and frozen for analysis of IL-8 by flow cytometry. All experiments included control wells treated with *E*. *coli* K235 LPS (1 ug/ml) (Sigma Chemical Co., St. Louis MO) or buffer diluents of each antigen [5,6].

### Cytokine analysis

Cytokine concentrations were determined by multianalyte microsphere flow cytometry as previously detailed [5]. Samples were measured in duplicate and corrected for blank values. Final concentrations were corrected for inter-experiment differences in macrophage numbers. Additional details on analysis are included in (S2 Text).

### Statistical analysis

Demographic data were normally distributed and were analyzed with ANOVA (Fisher’s test) for multiple comparisons. Demographic data are expressed as means ± SEM (Tables 2 and 3). Induced cytokine data among groups were not normally distributed and are reported as median [interquartile range (IQR)], analyzed by Kruskal Wallis and Mann Whitney U rank tests. For all comparisons, a p value of <0.05 was considered significant. To confirm whether age, cumulative smoking and lung function were independent variables related to macrophage cytokine induction, regression analysis was performed. Association with categorical variables (sex, race) was analyzed by analysis of variance (ANOVA).

**Table 2:** Demographic characteristics: Total study participants. Data are expressed as mean±SEM.

	nonCOPD	COPD		
Characteristics	Group 1, Nonsmokers, n = 20	Group 2, Ex-smokers, n = 83	Group 3, Active smokers, n = 93	p value
Age (years), Mean ± SEM	57.3±3.1	67.2±1.0	56.9±0.9	p≤0.02 ^a^ ^,^ ^c^
Gender, number				
Male	10	61	70	p = 0.07
Female	10	22	23	
Race, number				
Caucasian	16	75	70	p = 0.09
African-American	4	8	23	
Pack-years smoking Mean ± SEM	0±0	52.1±2.7	51.0±2.6	p<0.0001 ^a^ ^,^ ^b^
FEV_1_ (L)Mean ± SEM	2.88±0.18	1.64±0.08	1.95±0.08	p<0.006 ^a^ ^,^ ^b^ ^,^ ^c^
FEV_1_ (% predicted)Mean + SEM	99.55±3.57	54.46±2.28	61.48±2.00	p<0.03 ^a^ ^,^ ^b^ ^,^ ^c^
FVC (L)Mean ± SEM	3.64±0.20	3.13±0.10	3.53±0.10	p<0.04 ^a^ ^,^ ^c^
FVC, % predicted Mean ± SEM	98.50±3.10	81.79±2.31	87.30±1.90	p<0.02 ^a^ ^,^ ^b^
FEV_1_/FVC Mean ± SEM	80.05±3.0	51.21±1.70	54.44±1.27	p<0.0001 ^a^ ^,^ ^b^
GOLD classification^#^		% (n)	% (n)	
1		12.0% (10)	17.2% (16)	
2		44.6% (37)	55.9% (52)	p = 0.14
3		30.1% (25)	19.4% (18)	
4		13.2% (11)	7.5% (7)	

^a^ p≤0.05—nonsmokers vs. ex-smokers

^b^ p≤0.05—nonsmokers vs. active smoker

^c^ p≤0.05—ex-smokers vs. active smokers

^#^ GOLD classification is as detailed in the Global Initiative for Chronic Obstructive Lung Disease [24].

**Table 3:** Demographic characteristics: Bronchoscopy participants. Data are expressed as mean±SEM.

	nonCOPD	COPD		
Characteristics	Group1, Nonsmokers, n = 20	Group 2, Ex-smokers, n = 32	Group 3, Active smokers, n = 64	p value
Age (years), Mean ± SEM	57.3±3.1	64.5±1.3	56.5±1.2	p≤0.02 ^a^ ^,^ ^c^
Gender, number				
Male	10	23	46	p = 0.16
Female	10	9	18	
Race, number				
Caucasian	16	30	41	p = 0.02 ^c^
African-American	4	2	23	
Pack-years smokingMean ± SEM	0±0	54.6±4.4	48.4±3.1	p<0.0001 ^a^ ^,^ ^b^
FEV_1_ (L)Mean ± SEM	2.88±0.18	1.86±0.08	2.01±0.09	p<0.0001 ^a^ ^,^ ^b^
FEV_1_ (% predicted)Mean + SEM	99.55±3.57	61.64±2.54	64.90±2.19	p<0.0001 ^a^ ^,^ ^b^
FVC (L),Mean ± SEM	3.64±0.20	3.27±0.87	3.55±0.12	p>0.15
FVC, % predictedMean ± SEM	98.50±3.10	83.56±3.37	90.84±2.13	p≤0.05 ^a^ ^,^ ^c^
FEV_1_/FVCMean ± SEM	80.05±3.0	56.77±2.05	55.87±1.39	p<0.0001 ^a^ ^,^ ^b^
GOLD classification^#^		% (n)	% (n)	
1		12.5% (4)	20.3% (13)	
2		59.4% (19)	59.4% (38)	p = 0.55
3		28.1% (9)	20.3% (13)	

^a^ p≤0.05—nonsmokers vs. COPD ex-smokers

^b^ p≤0.05—nonsmokers vs. COPD active smokers

^c^ p≤0.05 –COPD ex-smokers vs. COPD active smokers

^#^ GOLD classification is as detailed in the Global Initiative for Chronic Obstructive Lung Disease [24].

For all comparisons of biologic outcomes elated to any SNP, carriers of the specific polymorphism were compared to all non-carriers of that polymorphism.

By design, the study was a genotypic association test of the COPD subgroups. Frequencies of all polymorphisms were tested in the control groups for consistency with Hardy-Weinberg equilibrium via the use of the Hardy Weinberg package developed for the R statistical programming environment [27]. Comparison was performed with in-sample frequencies via the exact method due to low number of subjects with double minor alleles. Complete data is included in (S2 Table).

## Results

### Recruitment of participants

Recruitment efforts enrolled 196 volunteers, including 141 men and 55 women. Group 1 (“healthy nonsmokers”) included 20 participants; Group 2 (“COPD ex-smokers”) included 83 participants; Group 3 (“COPD active smokers”) included 93 participants. Blood samples were obtained from all participants for TLR SNP analysis. Demographic data are presented in Tables 2 and 3.

Of 196 volunteers, 116 (79 men and 37 women) met inclusion criteria to safely undergo bronchoscopy. Demographic data for bronchoscopy participants are presented separately (Table 3). For all participants, as well as bronchoscopy participants, group disparities existed in spirometric parameters, cumulative pack-years and age. Therefore, regression analyses were used to determine the independent impact of each on immunologic outcomes.

### Frequency of TLR SNPs

To determine the relative frequency, each TLR polymorphism was statistically compared among groups of all participants (Table 4). Single nucleotide polymorphisms for TLR2(Arg753Gln), TLR4(Asp299Gly) and TLR4(Thr399Ile) were present in relative low frequency in all groups. Incidence of TLR polymorphisms by genotype is also given for TLR9(T1237C) and for TLR9(T1486C), as well as polymorphism frequency defined by minor allele frequency (S3 Table). Incidence and frequency for TLR2 and TLR4 SNPs is also in S4 Table. Our population had no homozygous minor alleles for TLR2(Arg753Gln) and TLR4(Asp299Gly) for any groups and only two donors homozygous for minor alleles for TLR4(Thr399Ile).

**Table 4:** Frequency of TLR polymorphisms. Frequency is shown of each TLR polymorphism (SNP) compared with wildtype (w/t) among each group of the total study population^#^. Statistical p values were determined by chi-square test.

TLR SNP	nonCOPD		COPD					
	Group 1 (n = 19)^a^		Group 2 (n = 74)^b^		Group 3 (n = 85)^c^		p value^d^	p value COPD vs. nonCOPD
	w/t	SNP	w/t	SNP	w/t	SNP		
	% (n)	% (n)	% (n)	% (n)	% (n)	% (n)		
TLR2 Arg753Gln	89.5 (17)	10.5 (2)	89.2 (66)	10.8 (8)	95.2 (80)	4.8 (4)	0.34	0.65
TLR4 Asp299Gly	94.7 (18)	5.3 (1)	93.2 (69)	6.8 (5)	92.9 (79)	7.1 (6)	0.96	0.79
TLR4 Thr399Ile	94.7 (18)	5.3 (1)	93.3 (70)	6.7 (5)	94.1 (80)	5.9 (5)	0.98	0.88
TLR9 T1237C	75 (15)	25 (5)	71.6 (53)	28.4 (21)	58.1 (50)	41.9 (36)	0.21	0.32
TLR9 T1486C	36.8 (7)	63.2 (12)	39.2 (29)	60.8 (45)	44.6 (37)	55.4 (46)	0.53	0.88

^a^n = 20 for Group 1, TLR9(T1237C).

^b^n = 75 for Group 2, TLR4(Thr399Ile).

^c^n = 86 for Group 3, TLR9(T1237C); n = 83 for TLR9(T1486C); n = 84 for TLR2(Arg753Gln)

^d^p value for comparison of all three groups

^#^DNA quantity was insufficient for analysis for some members of each group

Polymorphisms for both TLR9 SNPs were present in higher frequency. TLR9(T1237C) was expressed in 25% of healthy nonsmokers, 28.4% of COPD ex-smokers and 41.9% of COPD active smokers (Table 4). TLR9(T1486C) was expressed in 63.2% of nonsmokers, 60.8% of COPD ex-smokers and 55.4% of COPD active smokers. Eleven (8.9%) participants with TLR9(T1237C) and 23 (22.5%) with TLR9(T1486C) were heterozygous. No TLR SNPs had significantly greater carriage in any group including when COPD active and ex-smokers were combined and compared with healthy controls. Comparable statistical associations were determined among individuals of each group undergoing bronchoscopy (Table 5). All SNPs among control observations were within Hardy-Weinberg equilibrium (S2 Table).

**Table 5:** Frequency of TLR polymorphisms. Frequency is shown of each TLR polymorphism (SNP) compared with wildtype (w/t) among each group of bronchoscopy participants. Statistical comparisons are as indicated in Table 4.

TLR SNP	nonCOPD			COPD					
	Group 1 (n = 19)^a^			Group 2 (n = 31)		Group 3 (n = 62)^b^		p value^c^	p value COPD vs. nonCOPD
	w/t		SNP	w/t	SNP	w/t	SNP		
	% (n)		% (n)	% (n)	% (n)	% (n)	% (n)		
TLR2 Arg753Gln	89.5 (17)	10.5 (2)		90.3 (28)	9.7 (3)	95.2 (59)	4.8 (3)	0.57	0.53
TLR4 Asp299Gly	94.7 (18)	5.3 (1)		93.5 (29)	6.5 (2)	95.2 (60)	4.8 (3)	0.95	0.98
TLR4 Thr399Ile	94.7 (18)	5.3 (1)		90.3 (28)	9.7 (3)	91.9 (57)	8.1 (5)	0.94	0.79
TLR9 T1237C	75 (15)	25 (5)		67.7 (21)	32.3 (10)	56.5 (35)	43.5 (27)	0.20	0.20
TLR9 T1486C	36.8 (7)	63.2 (12)		54.8 (17)	45.2 (14)	38.7 (24)	61.2 (38)	0.30	0.53

^a^n = 20 for Group 1, TLR9(T1237C).

^b^n = 63 for Group 3, TLR4(Asp299Gly).

^c^p value for comparison of all three groups

### Association of TLR SNPs with IL-8 induction

To investigate the potential contribution of each TLR SNP to impaired innate immune responses of alveolar macrophages, induced IL-8 responses were compared among groups. TLR2(Arg753Gln), TLR4(Asp299Gly) and TLR4(Thr399Ile) polymorphisms held no significant correlations with IL-8 induction. Although statistically significant impairment of MC-induced IL-8 among active smokers was noted for TLR2(Arg753Gln) (p = 0.03), frequency of this polymorphism was limited to three participants.

**Fig 2:**
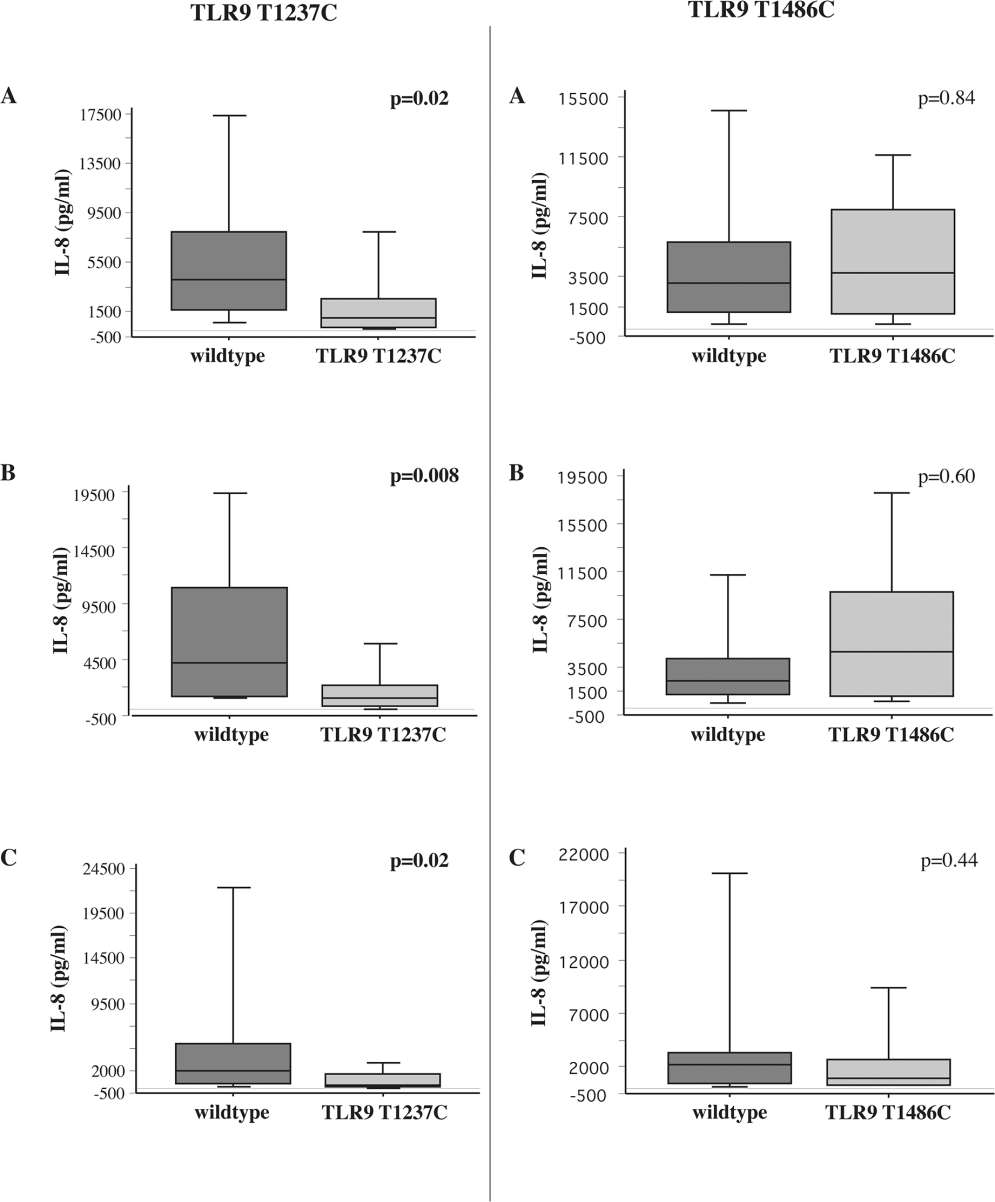
Association of TLR9 SNPs and bacterial induction of COPD ex-smoker alveolar macrophage IL-8. Supernatant IL-8 concentrations of alveolar macrophages, obtained from ex-smokers with COPD, incubated with nontypeable *Haemophilus influenzae* 11P6H1 (Panels A), *Moraxella catarrhalis* 6P29B1 (Panels B) and *Streptococcus pneumoniae* 25P55S1 (Panels C), were measured at 6 hours. Analysis is shown for TLR9 wildtype (dark shading) compared with TLR9 (T1237C) (light shading) on left panels. Analysis for TLR9 wildtype (dark shading) compared with TLR9 (T1486C) (striped shading) is shown in right panels. Results are shown as box plots for each group. Each box encompasses the 25^th^ to 75^th^ interquartile range, with the horizontal line in each box representing median values. Each vertical bar encompasses the 10^th^ to 90^th^ percentile ranges. Values correspond with data given in Table 6.

**Fig 3:**
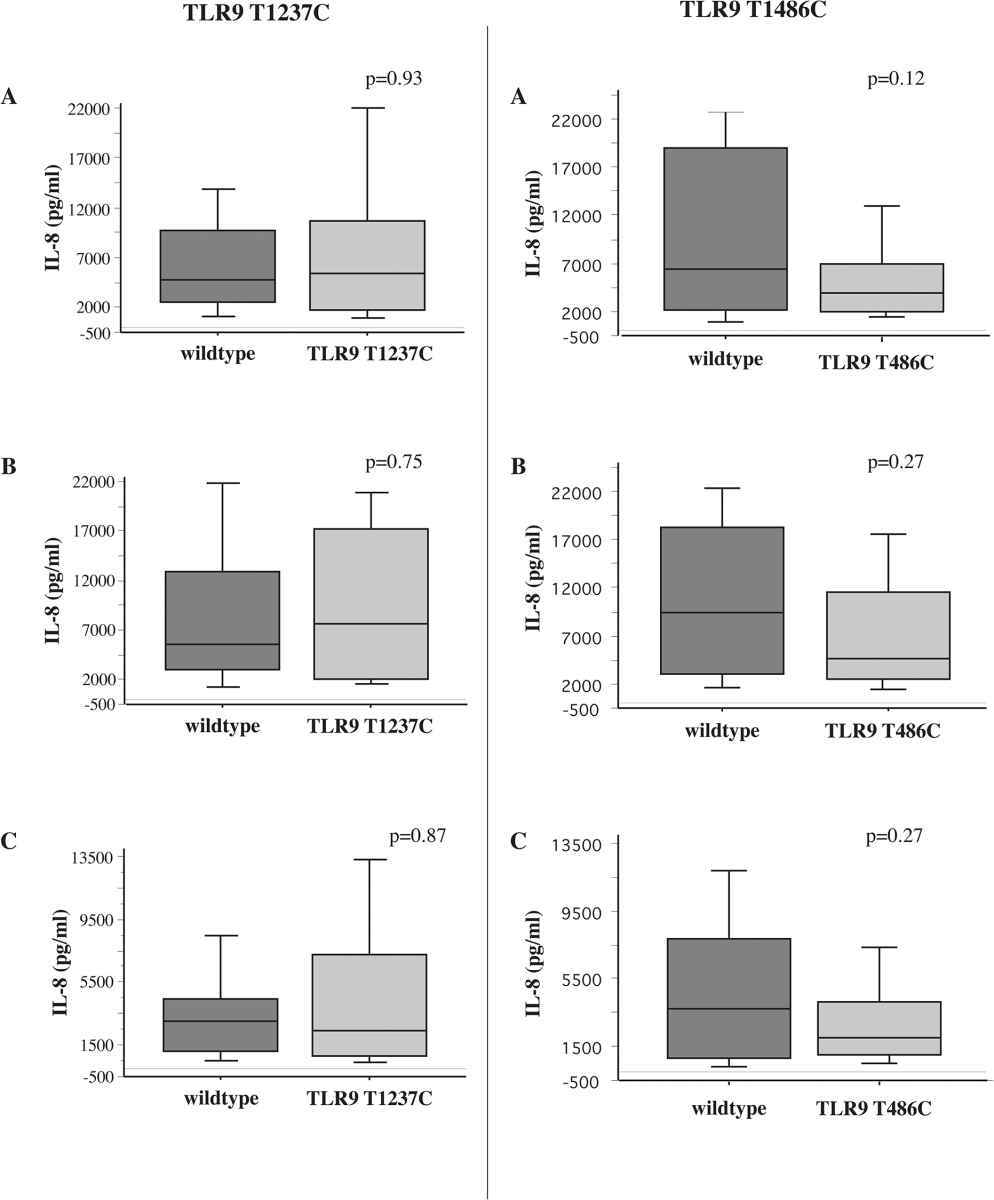
Association of TLR9 SNPs and bacterial induction of COPD active smoker alveolar macrophage IL-8. Supernatant IL-8 concentrations of alveolar macrophages, obtained from active smokers with COPD, incubated with the same three bacterial strains detailed in Fig 3, were measured. Shadings for wildtype, TLR9 (T1237C), and for TLR9 (T1486C) are as detailed in Fig 2.

Our study population had a higher frequency of both TLR9 SNPs, lending both to more detailed study. For all comparisons, the wildtype for each SNP is defined as the group that did not express the genotype of the given SNP. For example, for TLR9(T1237C), the wildtype group consists of all those that did not express TLR9(T1237C). Among ex-smokers with COPD, TLR9(T1237C) expression directly correlated with impaired IL-8 responsiveness of alveolar macrophages to NTHI (p = 0.02), MC (p = 0.008) and SP (p = 0.02) compared with wildtype expression (Fig 2, Table 6). In contrast, IL-8 responses of alveolar macrophages of COPD ex-smokers expressing TLR9(T1486C) were not statistically different from their wildtype counterparts (Fig 2). IL-8 responses for nonsmokers (not shown) and COPD active smokers did not correlate with carriage of TLR9(T1486C) or TLR9(T1237C) (Fig 3, Table 6). No TLR SNPs in this study had an association with TNF-alpha responses. These data are additionally arranged for each bacterial strain from all three cohorts and by log_10_IL-8 values (S5 and S6 Tables).

**Table 6:** Alveolar macrophage IL-8 (pg/ml) induction of alveolar macrophages expressing wildtype (w/t), TLR9 (T1237C) and TLR9 (T1486C). IL-8 values are expressed as median [IQR] and correspond with data of Figs 2 and 3.

Groups	NTHI				MC				SP			
	w/t	TLR9 (T1237C)	w/t	TLR9 (T1486C)	w/t	TLR9 (T1237C)	w/t	TLR9 (T1486C)	w/t	TLR9 (T1237C)	w/t	TLR9 (T1486C)
1 (nonsmokers)	7200 [9532]	2190 [6189]	5050 [9171]	643 [9652]	4225 [4319]	1815 [4910]	3405 [3159]	4313 [6800]	2005 [2040]	720 [1526]	1515 [2359]	2050 [2134]
2 (COPD ex-smokers)	4105 [6280]	1030* [2362]	3005 [4690]	3764 [6910]	4220 [9700]	1125* [1876]	2400 [2967]	4800 [8673]	1980 [4399]	438* [1489]	2160 [2903	965 [2306]
3 (COPD active smokers)	4730 [7170]	5400 [8880]	6375 [16755]	3980 [5018]	5510 [9915]	7650 [15090]	9400 [15317]	4618 [8958]	3058 [3305]	2400 [6434]	3735 [7032]	2038 [3050]

*p<0.05- TLR9 SNP vs. w/t

### Relationship of TLR9 SNPs with pulmonary function

**Fig 4:**
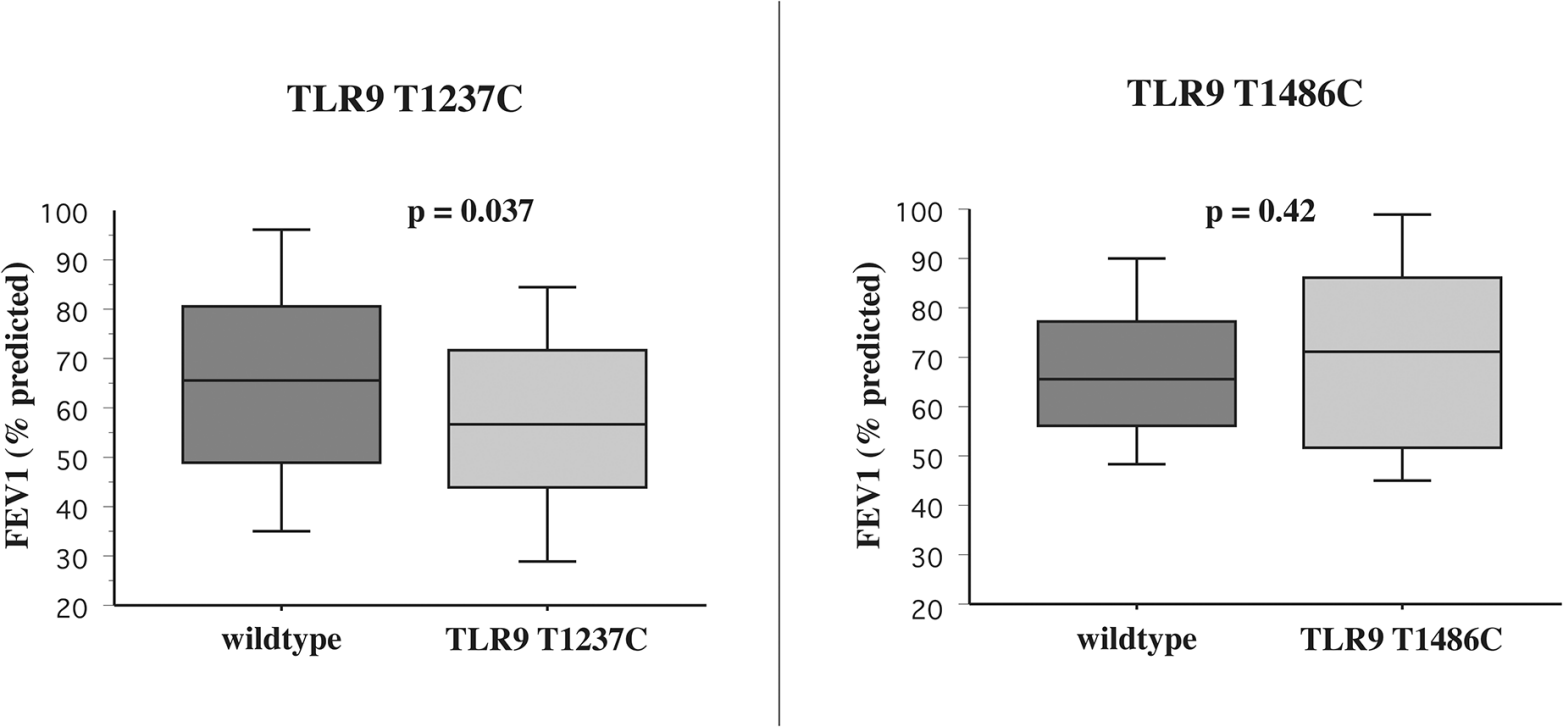
TLR9 SNPs and FEV_1_%predicted. Correlation of FEV_1_%predicted and expression of TLR9 wildtype (dark shading) compared with (T1237C) (light shading) is shown in left panel. Correlation of FEV_1_%predicted and expression of TLR9 wildtype (dark shading) compared with TLR9 (T1486C) (striped shading) is shown in right panel. Results are given for all COPD participants.

To determine the relationship of TLR9 SNPs with lung function, comparison was made with FEV_1_%predicted for all participants (Fig 4). FEV_1_%predicted was significantly diminished among TLR9(T1237C) participants compared with wildtype (p = 0.037). In contrast, FEV_1_%predicted was no different among participants with TLR9(T1486C) expression compared with wildtype (p = 0.42). FEV_1_%predicted was also not statistically different for TLR2(Arg753Gln), TLR4(Thr399Ile) and TLR4(Asp299Gly) carriage compared with wildtype counterparts.

### Regression analyses

Because demographic differences existed between groups (Tables 2 and 3), regression analyses were performed to determine the independent relationship of age, cumulative smoking, lung function (FEV_1_%predicted), race and sex with alveolar macrophage cytokine induction for each group. For this analysis, a p value of <0.05 was considered significant. Tables with complete data (p values, r^2^ values) are included in (S1 Table). In all analyses of alveolar macrophages, age, cumulative smoking, lung function, race and sex were not independent determinants for alveolar macrophage cytokine responses for any respiratory pathogen (p>0.05 for each). Thus, the association of IL-8 induction was independent of demographic variables. Further analysis also did not demonstrate an independent association of demographic variables other than FEV_1_%predicted (Tables 2 and 3) with TLR9(T1237C) carriage.

## Discussion

This study is the first to identify a relationship of dysfunctional COPD innate immune responses with a specific TLR9 polymorphism and the first to reveal a conspicuous association between expression of TLR9(T1237C) and severity of COPD. The contribution of TLR SNPs to human disease has only recently been explored. A major consideration has been the possibility of attenuated responses of TLR SNPs to TLR ligands [11,12], although focus has included enhanced susceptibility of TLR2 and TLR4 polymorphisms to a wide range of pathogens [25,26].

Correlations with TLR polymorphisms have been established to varying degrees, for several remarkably diverse inflammatory disease states [11,22]. Increased frequency of TLR4(Asp299Gly) occurred in Crohn’s disease and ulcerative colitis, indicating a potential role for deficient host-bacterial interactions underlying inflammatory bowel disease [28,29]. TLR9(T1237C) expression also has increased prevalence in Crohn’s disease, particularly among those with NOD2 mutations and IL23R variants [30].

Recent studies on the frequency of TLR SNPs in COPD have yielded diverse results. For example, Sabroe *et al*. did not find a change in frequency of TLR4(Asp299Gly) in COPD, while a later investigation described decreased frequency of this allele in COPD [15,17]. TLR4 polymorphism (rs11536889) was also associated with COPD in a Japanese population [31]. Others have found increased frequency of TLR4(T399I) and TLR9(T1486C) in COPD [16,18]. In contrast, TLR2(Arg753Gln) had no correlation with either COPD or unstable COPD [32]. These results do not necessarily conflict with ours, as most of these studies included a larger nonCOPD group, with less diverse ethnicity than our study was designed for. Rather, our study was designed to focus on the immunologic consequences of TLR SNPs in COPD. SNPs of the preceding studies were neither correlated with any immune dysfunction nor with severity of COPD.

TLR9 is the only TLR in humans that recognizes bacterial or viral single stranded DNA, discriminating between increased percent of unmethylated CpG dinucleotides [33]. TLR9 is localized in intracellular endosomal compartments where pathogen DNA must be degraded to single stranded DNA before interacting with TLR9 [32]. CpG-DNA binding to TLR9 initiates MyD88 recruitment leading to NF-kB activation [33,34]. Twenty SNPs of TLR9 have been described [35], of which some are expressed with relatively high frequency. For example, TLR9(T1237C) and (A2848G) each had greater than 10% frequency among European, African and Hispanic ethnic groups in the US [35]. Interestingly, TLR9(T1237C) was strongly associated with asthma among European-Americans [36], yet correlated poorly with asthma among Japanese. This may relate more to low level carriage of this allele among Japanese than to functional significance [33]. Despite an association with asthma, expression of five TLR9 SNPs, including TLR9(T1237C), failed to correlate with clinical atopy [37].

In addition, TLR9(T1237C) also had increased prevalence in allergic bronchopulmonary aspergillosis, while TLR4(Asp299Gly) had significant association with chronic cavitary pulmonary aspergillosis [19]. Expression of TLR9(T1237C) is also linked to relative risk for *H*. *pylori*-induced premalignant gastric disease and renal disease [34,38]. Although some of these associations suggest a potentially exaggerated inflammatory response associated with the TLR9(T1237C) allele, other studies of TLR SNPs are more consistent with the hyporesponsive profile of alveolar macrophage inflammatory responses to bacteria pathogens that we observed in COPD ex-smokers expressing TLR9(T1237C) [11,12]. Our findings of impaired IL-8 responsiveness to three separate respiratory bacteria, among TLR9(T1237C) expressers, indicates a broad range of PAMP recognition, a characteristic of TLR function. While diminished IL-8 induction with TLR9(T1237C) was pronounced among COPD ex-smokers, it was not apparent among active smokers. We suspect this to be due to redundancy of inflammatory mechanisms and the immunologic potential of environmental factors such as cigarette smoke to overwhelm this impairment, adding strong rationale for independent study of immunologic impairments of COPD in active and ex-smokers. The association of the TLR9(T1237C) allele with significantly lower FEV_1_%predicted values suggests that inability to mount a strong inflammatory response to respiratory pathogens, as occurred with TLR9(T1237C) expression, correlates with poorer long-term outcome. The precise significance of this association will require further study. Although induced IL-8 values are also lower among nonsmokers (Table 6), differences were not sufficient to achieve statistical significance. However, it is possible that with a greater sample size, macrophage hyporesponsiveness related to this SNP might be appreciated in groups other than COPD ex-smokers.

Recognition of endogenous bacteria by TLRs under steady state conditions may play a role in intestinal homeostasis, in which activation of TLRs by commensal microflora provides protection from injury [39]. Thus, if expression of specific TLR SNPs interferes with receptor-ligand contact, interactions might lead to an aberrant immune response, as occurs in Crohn’s disease [39]. Comparable disequilibrium between products of respiratory microflora and TLR SNPs in individuals with COPD might well underlie impaired innate immune dysfunction, leading to aberrant macrophage responses contributing to progression of COPD.

Our identification of a specific association of TLR9(T1237C) with COPD alveolar macrophage dysfunction may have been assisted by the relatively high frequency expression of this allele in our population. Nonetheless, TLR9(T1486C) had comparable relative frequency, yet had neither an association with impaired alveolar macrophage responses nor with severity of COPD. Thus, our findings appear pertinent to the specific polymorphism rather than to all TLR9 SNPs. While TLR9(T1486C) may have greater relative frequency in COPD [18], our investigation did not support a comparable immunologic role for this allele. The lower frequency of expression of other TLR SNPs in our study population does not preclude the contribution of other TLR polymorphisms to alveolar macrophage dysfunction in COPD. Just as Crohn’s disease is not limited to association with one TLR SNP, the lower frequency of other TLR SNPs in our study population does not preclude the contribution of other TLR polymorphisms to severity of COPD. In fact, a study of European subjects found two TLR2 SNPs to be associated with diminished FEV_1_, while two others were associated with increased FEV_1_ [40]. While this may relate to ethnic variation in our study population, it does not detract from associations with TLR9(T1237C) in our study. Our previous findings of defective COPD macrophage responses to TLR2 and TLR4 ligands [5] leave open the possibility that TLR2 and TLR4 SNPs might play a role in this setting, but may not have been present in high enough frequency in our study to determine an association.

In sum, our findings support a role for the TLR9(T1237C) allele as one component underlying dysfunctional innate immune responses of alveolar macrophages and progression of disease in COPD.

## Supporting Information

S1 Text Bacterial strains. (DOC)

S2 Text Cytokine analysis. (DOC)

S1 Table Regression analysis tables. (DOC)

S2 Table SNP variance from Hardy-Weinberg equilibrium. (DOCX)

S3 Table Incidence of TLR9 polymorphisms by genotype. (DOC)

S4 Table Incidence of TLR2 and 4 polymorphisms by genotype. (DOC)

S5 Table Alveolar macrophage IL-8 (pg/ml) induction of alveolar macrophages expressing wildtype (w/t), TLR9 (T1237C) and TLR9 (T1486C). (DOC)

S6 Table Alveolar macrophage log_10_ IL-8 (pg/ml) induction of alveolar macrophages expressing wildtype (w/t), TLR9 (T1237C) and TLR9 (T1486C). (DOC)
