# Supplementary material for: Impaired Innate COPD Alveolar Macrophage Responses and Toll-Like Receptor-9 Polymorphisms
Source: PLoS One. 2015 Sep 11;10(9):e0134209. doi: 10.1371/journal.pone.0134209 (PMC4567310; doi:10.1371/journal.pone.0134209)
Supplement: S1 Text — (DOC) [file pone.0134209.s001.doc]

**S1 Text:** **Bacterial strains**

Bacterial strains that were utilized included nontypeable *Haemophilus* *influenzae* 11P6H1; *Streptococcus pneumoniae* 25P55 and *Moraxella catarrhalis* 6P29B1. Each is well characterized and is fully viable in 8% antibody-depleted serum. All three bacterial strains were obtained from sputum from COPD patients and all were associated with COPD exacerbations. All elicited a systemic and/or mucosal antibody response to the infecting strain, following the COPD exacerbation and are thus more relevant to clinical studies of COPD than laboratory or type strains.
