# Supplementary material for: Impaired Innate COPD Alveolar Macrophage Responses and Toll-Like Receptor-9 Polymorphisms
Source: PLoS One. 2015 Sep 11;10(9):e0134209. doi: 10.1371/journal.pone.0134209 (PMC4567310; doi:10.1371/journal.pone.0134209)
Supplement: S2 Text — (DOC) [file pone.0134209.s002.doc]

**S2 Text: Cytokine analysis**

Cytokine concentrations were determined by multianalyte microsphere assay, analyzed by flow cytometry. Capture and detection antibody pairs directed against different noncompeting epitopes of their respective cytokine and recombinant protein standards for IL-8 and TNF-α, (R&D Systems (Minneapolis, MN) were covalently coupled to multi-analyte carboxylated microspheres (Luminex Corp., Austin, TX), each set of which has a unique proportion of red and orange fluorescent dyes. Cytokine capture antibodies were each coupled to a different microsphere bead set. Biotinylated detection antibodies for each cytokine, diluted in PBS-TBN, were added. Phycoerythrin-conjugated streptavidin (Caltag Laboratories, Burlingame, CA) was added to each well and the beads were resuspended and analyzed by flow cytometry (Luminex 100, Luminex Corp).
