# Supplementary material for: Impaired Innate COPD Alveolar Macrophage Responses and Toll-Like Receptor-9 Polymorphisms
Source: PLoS One. 2015 Sep 11;10(9):e0134209. doi: 10.1371/journal.pone.0134209 (PMC4567310; doi:10.1371/journal.pone.0134209)
Supplement: S1 Table — (DOC) [file pone.0134209.s003.doc]

**S1 Tables:**  **Regression analysis tables**

IL-8 induction from alveolar macrophages was analyzed for independent association with continuous variables (age, FEV_1_ %predicted and smoking pack-years) by regression analysis. Association with categorical variables (sex, race) was analyzed by analysis of variants. Separate analyses were performed for induction by each bacterial pathogen and for ex-smokers, active smokers and nonsmokers.

Ex-smokers

*M. catarrhalis*

| Independent variable (continuous) | p value | r^2^ |  | Independent variable (categorical) | p value |
| --- | --- | --- | --- | --- | --- |
| Age | 0.604 | 0.009 |  | Sex | 0.346 |
| FEV_1_ %predicted | 0.080 | 0.100 |  | Race | 0.465 |
| Pack years | 0.230 | 0.046 |  | | |

*Nontypeable H. influenzae*

| Independent variable (continuous) | p value | r^2^ |  | Independent variable (categorical) | p value |
| --- | --- | --- | --- | --- | --- |
| Age | 0.699 | 0.005 |  | Sex | 0.794 |
| FEV_1_ %predicted | 0.164 | 0.032 |  | Race | 0.418 |
| Pack years | 0.268 | 0.009 |  | | |

*S. pneumoniae*

| Independent variable (continuous) | p value | r^2^ |  | Independent variable (categorical) | p value |
| --- | --- | --- | --- | --- | --- |
| Age | 0.975 | 0.006 |  | Sex | 0.426 |
| FEV_1_ %predicted | 0.234 | 0.045 |  | Race | 0.292 |
| Pack years | 0.092 | 0.089 |  | | |

*Active smokers*

*M. catarrhalis*

| Independent variable (continuous) | p value | r^2^ |  | Independent variable (categorical) | p value |
| --- | --- | --- | --- | --- | --- |
| Age | 0.050 | 0.064 |  | Sex | 0.061 |
| FEV_1_ %predicted | 0.702 | 0.002 |  | Race | 0.296 |
| Pack years | 0.117 | 0.040 |  | | |

*Nontypeable H. influenzae*

| Independent variable (continuous) | p value | r^2^ |  | Independent variable (categorical) | p value |
| --- | --- | --- | --- | --- | --- |
| Age | 0.214 | 0.009 |  | Sex | 0.462 |
| FEV_1_ %predicted | 0.823 | 0.001 |  | Race | 0.414 |
| Pack years | 0.348 | 0.014 |  | | |

*S. pneumoniae*

| Independent variable (continuous) | p value | r^2^ |  | Independent variable (categorical) | p value |
| --- | --- | --- | --- | --- | --- |
| Age | 0.192 | 0.028 |  | Sex | 0.142 |
| FEV_1_ %predicted | 0.130 | 0.037 |  | Race | 0.976 |
| Pack years | 0.816 | 0.001 |  | | |

*Nonsmokers*

*M. catarrhalis*

| Independent variable (continuous) | p value | r^2^ |  | Independent variable (categorical) | p value |
| --- | --- | --- | --- | --- | --- |
| Age | 0.244 | 0.001 |  | Sex | 0.810 |
| FEV_1_ %predicted | 0.542 | 0.021 |  | Race | 0.688 |

*Nontypeable H. influenzae*

| Independent variable (continuous) | p value | r^2^ |  | Independent variable (categorical) | p value |
| --- | --- | --- | --- | --- | --- |
| Age | 0.609 | 0.015 |  | Sex | 0.528 |
| FEV_1_ %predicted | 0.122 | 0.079 |  | Race | 0.657 |

*S. pneumoniae*

| Independent variable (continuous) | p value | r^2^ |  | Independent variable (categorical) | p value |
| --- | --- | --- | --- | --- | --- |
| Age | 0.909 | 0.001 |  | Sex | 0.850 |
| FEV_1_ %predicted | 0.561 | 0.019 |  | Race | 0.732 |
