# Supplementary material for: Impaired Innate COPD Alveolar Macrophage Responses and Toll-Like Receptor-9 Polymorphisms
Source: PLoS One. 2015 Sep 11;10(9):e0134209. doi: 10.1371/journal.pone.0134209 (PMC4567310; doi:10.1371/journal.pone.0134209)
Supplement: S2 Table — (DOCX) [file pone.0134209.s004.docx]

**S2 Table**: **SNP variance from Hardy-Weinberg equilibrium.** Variance is shown of each TLR polymorphism from the control group of the total study population. Statistical p values were determined by exact test.

| TLR2  Arg753Gln  G is ancestral allele | Observed Genotype Counts, n (% of row total) | | |  | Observed Allele Counts, n (%) | | Exact test |
| --- | --- | --- | --- | --- | --- | --- | --- |
| rs5743708*  MAF=0.0068* | G/G | G/A | A/A | total | G | A | p-value |
| nonCOPD nonsmoker | 17 (89) | 2 (11) | 0 | 19 | 36 (95) | 2 (5) | 1 |

| TLR4  Asp299Gly  A is ancestral allele | Observed Genotype Counts, n (% of row total) | | |  | Observed Allele Counts, n (%) | | Exact test |
| --- | --- | --- | --- | --- | --- | --- | --- |
| rs4986790*  MAF=0.0599* | A/A | A/G | G/G | total | A | G | p-value |
| nonCOPD nonsmoker | 18 (95) | 1 (5) | 0 | 19 | 17 (97) | 1 (3) | 1 |

| TLR4  Thr399Ile  C is ancestral allele | Observed Genotype Counts, n (% of row total) | | |  | Observed Allele Counts, n (%) | | Exact test |
| --- | --- | --- | --- | --- | --- | --- | --- |
| rs4986791*  MAF=0.0407* | C/C | C/T | T/T | total | C | T | p-value |
| nonCOPD nonsmoker | 18 (95) | 1 (5) | 0 | 19 | 37 (97) | 1 (3) | 1 |

| TLR9  T1237C  C is ancestral allele | Observed Genotype Counts, n (% of row total) | | |  | Observed Allele Counts, n (%) | | Exact test |
| --- | --- | --- | --- | --- | --- | --- | --- |
| rs5743836*  MAF=0.1725* | C/C | C/T | T/T | total | C | T | p-value |
| nonCOPD nonsmoker | 15 (75) | 3 (15) | 2 (10) | 20 | 33 (82) | 7 (18) | 0.07 |

| TLR9  T1486C  C is ancestral allele | Observed Genotype Counts, n (% of row total) | | |  | Observed Allele Counts, n (%) | | Exact test |
| --- | --- | --- | --- | --- | --- | --- | --- |
| rs187084*  MAF=0.3776* | C/C | C/T | T/T | total | C | T | p-value |
| nonCOPD nonsmoker | 7 (37) | 9 (47) | 3 (16) | 19 | 23 (61) | 15 (39) | 1 |

* SNP designation and minor allele frequency (MAF) of reference genomes are from the Single Nucleotide Polymorphism Database (dbSNP).
