# Supplementary material for: Impaired Innate COPD Alveolar Macrophage Responses and Toll-Like Receptor-9 Polymorphisms
Source: PLoS One. 2015 Sep 11;10(9):e0134209. doi: 10.1371/journal.pone.0134209 (PMC4567310; doi:10.1371/journal.pone.0134209)
Supplement: S3 Table — (DOC) [file pone.0134209.s005.doc]

**S3 Tables. Incidence of TLR9 polymorphisms by genotype.**

**S3a Table.** Incidence is shown of each TLR9 polymorphism, including homozygous minor alleles, among each group of the total study population for TLR9 (T1237C) and for TLR9 (T1486C). Statistical p values were determined by chi-square (χ^2^) test.

| TLR SNP | nonCOPD nonsmoker | | | COPD ex-smoker | | | COPD active smoker | | |  |  |
| --- | --- | --- | --- | --- | --- | --- | --- | --- | --- | --- | --- |
|  | TT  n(%) | TC  n(%) | CC  n(%) | TT  n(%) | TC  n(%) | CC  n(%) | TT  n(%) | TC  n(%) | CC  n(%) | p value | p value  COPD vs. nonCOPD |
| TLR9 T1237C | 15 (75) | 3  (15) | 2  (10) | 53 (71.6) | 18 (24.3) | 3  (4.1) | 50 (58.1) | 30 (34.9) | 6  (7) | 0.24 | 0.32 |
| *Frequency | 0.18 | | | 0.16 | | | 0.24 | | |  | |
| TLR9 T1486C | 7  (36.8) | 9 (47.4) | 3 (15.8) | 29 (39.2) | 38 (51.4) | 7  (9.5) | 37 (44.6) | 33 (39.8) | 13 (15.7) | 0.59 | 0.88 |
| *Frequency | 0.39 | | | 0.35 | | | 0.36 | | |  | |

# S3b Table. Incidence is shown of each TLR polymorphism among each group of participants who underwent BAL. Statistical comparisons are as indicated in S5a Table.

| TLR SNP | nonCOPD nonsmoker | | | COPD ex-smoker | | | COPD active smoker | | |  |  |
| --- | --- | --- | --- | --- | --- | --- | --- | --- | --- | --- | --- |
|  | TT  n(%) | TC  n(%) | CC  n(%) | TT  n(%) | TC  n(%) | CC  n(%) | TT  n(%) | TC  n(%) | CC  n(%) | p value | p value  COPD vs. nonCOPD |
| TLR9 T1237C | 15 (75) | 3  (15) | 2  (10) | 21 (67.7) | 7 (22.6) | 3 (9.7) | 35 (56.5) | 23 (37.1) | 4  (6.5) | 0.36 | 0.30 |
| *Frequency | 0.18 | | | 0.21 | | | 0.25 | | |  | |
| TLR9 T1486C | 7 (36.8) | 9 (47.4) | 3 (15.8) | 17 (54.8) | 12 (38.7) | 2 (6.5) | 24 (39.3) | 27 (44.3) | 10 (16.4) | 0.53 | 0.82 |
| *Frequency | 0.39 | | | 0.26 | | | 0.38 | | |  | |

*Frequency of polymorphism defined by minor allele frequency: (CC x 2) + TC

Total x 2
