# Supplementary material for: Impaired Innate COPD Alveolar Macrophage Responses and Toll-Like Receptor-9 Polymorphisms
Source: PLoS One. 2015 Sep 11;10(9):e0134209. doi: 10.1371/journal.pone.0134209 (PMC4567310; doi:10.1371/journal.pone.0134209)
Supplement: S4 Table — (DOC) [file pone.0134209.s006.doc]

# S4 Tables. Incidence of TLR2 and 4 polymorphisms by genotype.

| TLR SNP | nonCOPD nonsmoker | | | COPD ex-smoker | | | COPD active smoker | | |  |  |
| --- | --- | --- | --- | --- | --- | --- | --- | --- | --- | --- | --- |
|  | Arg Arg  (n) | Arg Gln  (n) | Gln Gln  (n) | Arg Arg  (n) | Arg Gln  (n) | Gln Gln  (n) | Arg Arg  (n) | Arg Gln  (n) | Gln Gln  (n) | p value | p value  COPD vs. nonCOPD |
| TLR2 Arg753Gln | 17 | 2 | 0 | 66 | 8 | 0 | 80 | 4 | 0 | 0.34 | 0.65 |

# S4a Table. Incidence is shown by genotype of each TLR2 and 4 polymorphism among each group of the total study population. Statistical comparisons were determined by chi-square (χ^2^) test.

| TLR SNP | nonCOPD nonsmoker | | | COPD ex-smoker | | | COPD active smoker | | |  |  |
| --- | --- | --- | --- | --- | --- | --- | --- | --- | --- | --- | --- |
|  | Thr Thr  (n) | Thr Ile  (n) | Ile Ile  (n) | Thr Thr  (n) | Thr Ile  (n) | Ile Ile  (n) | Thr Thr  (n) | Thr Ile  (n) | Ile Ile  (n) | p value | p value  COPD vs. nonCOPD |
| TLR4 Thr399Ile | 18 | 1 | 0 | 70 | 5 | 1 | 80 | 5 | 1 | 0.92 | 0.73 |

| TLR SNP | nonCOPD nonsmoker | | | | COPD ex-smoker | | | COPD active smoker | | |  |  |
| --- | --- | --- | --- | --- | --- | --- | --- | --- | --- | --- | --- | --- |
|  | Asp Asp  (n) | | Asp Gly  (n) | Gly Gly  (n) | Asp Asp  (n) | Asp Gly  (n) | Gly Gly  (n) | Asp Asp  (n) | Asp Gly  (n) | Gly Gly  (n) | p value | p value  COPD vs. nonCOPD |
| TLR4 Asp299Gly | 18 | 1 | | 0 | 69 | 5 | 0 | 79 | 6 | 0 | 0.96 | 0.79 |

# S4b Table. Incidence is shown by genotype of each TLR2 and 4 polymorphism among each group of participants who underwent BAL. Statistical comparisons are as indicated in S6a Table.

| TLR SNP | nonCOPD nonsmoker | | | COPD ex-smoker | | | COPD active smoker | | |  |  |
| --- | --- | --- | --- | --- | --- | --- | --- | --- | --- | --- | --- |
|  | Thr Thr  (n) | Thr Ile  (n) | Ile Ile  (n) | Thr Thr  (n) | Thr Ile  (n) | Ile Ile  (n) | Thr Thr  (n) | Thr Ile  (n) | Ile Ile  (n) | p value | p value  COPD vs. nonCOPD |
| TLR4 Thr399Ile | 18 | 1 | 0 | 28 | 2 | 1 | 57 | 4 | 1 | 0.86 | 0.63 |

| TLR SNP | nonCOPD nonsmoker | | | | COPD ex-smoker | | | COPD active smoker | | |  |  |
| --- | --- | --- | --- | --- | --- | --- | --- | --- | --- | --- | --- | --- |
|  | Asp Asp  (n) | | Asp Gly  (n) | Gly Gly  (n) | Asp Asp  (n) | Asp Gly  (n) | Gly Gly  (n) | Asp Asp  (n) | Asp Gly  (n) | Gly Gly  (n) | p value | p value  COPD vs. nonCOPD |
| TLR4 Asp299Gly | 18 | 1 | | 0 | 29 | 2 | 0 | 59 | 3 | 0 | 0.95 | 0.98 |

| TLR SNP | nonCOPD nonsmoker | | | COPD ex-smoker | | | COPD active smoker | | |  |  |
| --- | --- | --- | --- | --- | --- | --- | --- | --- | --- | --- | --- |
|  | Arg Arg  (n) | Arg Gln  (n) | Gln Gln  (n) | Arg Arg  (n) | Arg Gln  (n) | Gln Gln  (n) | Arg Arg  (n) | Arg Gln  (n) | Gln Gln  (n) | p value | p value  COPD vs. nonCOPD |
| TLR2 Arg753Gln | 17 | 2 | 0 | 28 | 3 | 0 | 59 | 3 | 0 | 0.57 | 0.53 |
