# Supplementary material for: Impaired Innate COPD Alveolar Macrophage Responses and Toll-Like Receptor-9 Polymorphisms
Source: PLoS One. 2015 Sep 11;10(9):e0134209. doi: 10.1371/journal.pone.0134209 (PMC4567310; doi:10.1371/journal.pone.0134209)
Supplement: S5 Table — (DOC) [file pone.0134209.s007.doc]

|  | nonsmokers | COPD  ex-smokers | COPD active smokers | nonsmokers | COPD  ex-smokers | COPD active smokers | nonsmokers | COPD  ex-smokers | COPD active smokers |
| --- | --- | --- | --- | --- | --- | --- | --- | --- | --- |
| Wildtype | 7200  [9532] | 4105  [6280] | 4730 [7170] | 4225  [4319] | 4220 [9700] | 5510 [9915] | 2005  [2040] | 1980  [4399] | 3058 [3305] |
|  |  |  |  |  |  |  |  |  |  |
| TLR9  (T1237C) | 2190  [6189] | 1030*****  [2362] | 5400 [8880] | 1815  [4910] | 1125*****  [1876] | 7650 [15090] | 720  [1526] | 438*****  [1489] | 2400  [6434] |
|  |  |  |  |  |  |  |  |  |  |
| TLR9  (T1486C) | 6435  [9652] | 3764  [6910] | 3980  [5018] | 4313  [6800] | 4800  [8673] | 4618  [8958] | 2050  [2134] | 965  [2306] | 2038  [3050] |

**S5 Table:** **Alveolar macrophage IL-8 (pg/ml) induction of alveolar macrophages expressing wildtype (w/t), TLR9 (T1237C) and TLR9 (T1486C).** Data is arranged for each bacterial strain from all three groups. IL-8 values are expressed as median [IQR].

*****p<0.05- TLR9 SNP vs. w/t
