# Supplementary material for: Impaired Innate COPD Alveolar Macrophage Responses and Toll-Like Receptor-9 Polymorphisms
Source: PLoS One. 2015 Sep 11;10(9):e0134209. doi: 10.1371/journal.pone.0134209 (PMC4567310; doi:10.1371/journal.pone.0134209)
Supplement: S6 Table — (DOC) [file pone.0134209.s008.doc]

**S6 Table:** **Alveolar macrophage log_10_ IL-8 (pg/ml) induction of alveolar macrophages expressing wildtype (w/t), TLR9 (T1237C) and TLR9 (T1486C).** Log_10_ IL-8 values are expressed as median [IQR].

| Groups | NTHI | | | MC | | | SP | | |
| --- | --- | --- | --- | --- | --- | --- | --- | --- | --- |
|  | w/t | TLR9 (T1237C) | TLR9 (T1486C) | w/t | TLR9 (T1237C) | TLR9 (T1486C) | w/t | TLR9 (T1237C) | TLR9 (T1486C) |
| 1 (nonsmokers) | 3.86 [0.62] | 3.34 [0.89] | 3.81  [0.86] | 3.63 [0.51] | 3.26  [0.79] | 3.63  [0.78] | 3.30  [0.62] | 2.86  [0.60] | 3.31  [0.68] |
|  |  |  |  |  |  |  |  |  |  |
| 2 (COPD ex-smokers) | 3.61 [0.68] | 3.01***** [0.96] | 3.57  [0.89] | 3.62 [0.96] | 3.05*****  [0.88] | 3.68  [0.93] | 3.30 [0.89] | 2.64*****  [0.94] | 2.99  [0.85] |
|  |  |  |  |  |  |  |  |  |  |
| 3 (COPD active smokers) | 3.68  [0.58] | 3.73  [0.78] | 3.60  [0.54] | 3.74 [0.63] | 3.88 [0.92] | 3.66  [0.65] | 3.49 [0.59] | 3.38  [0.96] | 3.31  [0.60] |

*****p<0.05- TLR9 SNP vs. w/t
